# Statistical matching for conservation science

**DOI:** 10.1111/cobi.13448

**Published:** 2019-12-24

**Authors:** Judith Schleicher, Johanna Eklund, Megan D. Barnes, Jonas Geldmann, Johan A. Oldekop, Julia P. G. Jones

**Affiliations:** ^1^ Department of Geography University of Cambridge Cambridge CB2 1QB U.K.; ^2^ Department of Geosciences and Geography, Helsinki Institute of Sustainability Science, Faculty of Science University of Helsinki P.O. Box 64 (Gustaf Hällströmin katu 2A), FI‐00014 Finland; ^3^ School of Biology The University of Queensland St Lucia QLD 4067 Australia; ^4^ Biodiversity and Conservation Science Department of Biodiversity Conservation and Attractions 6983 Western Australia Australia; ^5^ Conservation Science Group Department of Zoology University of Cambridge Downing Street Cambridge CB2 3EJ U.K.; ^6^ Global Development Institute University of Manchester Oxford Road Manchester M13 9PL U.K.; ^7^ College of Engineering and Environmental Sciences Bangor University Thoday Road, Deniol Road, LL57 2UW U.K.

**Keywords:** causal inference, conservation effectiveness, counterfactual, impact evaluation, spillover, spatial autocorrelation, autocorrelación espacial, consecuencias indirectas, efectividad de la conservación, evaluación de impacto, hipótesis de contraste, inferencia causal, 因果推论, 保护有效性, 溢出效应, 空间自相关, 反事实, 效果评估

## Abstract

The awareness of the need for robust impact evaluations in conservation is growing and statistical matching techniques are increasingly being used to assess the impacts of conservation interventions. Used appropriately matching approaches are powerful tools, but they also pose potential pitfalls. We outlined important considerations and best practice when using matching in conservation science. We identified 3 steps in a matching analysis. First, develop a clear theory of change to inform selection of treatment and controls and that accounts for real‐world complexities and potential spillover effects. Second, select the appropriate covariates and matching approach. Third, assess the quality of the matching by carrying out a series of checks. The second and third steps can be repeated and should be finalized before outcomes are explored. Future conservation impact evaluations could be improved by increased planning of evaluations alongside the intervention, better integration of qualitative methods, considering spillover effects at larger spatial scales, and more publication of preanalysis plans. Implementing these improvements will require more serious engagement of conservation scientists, practitioners, and funders to mainstream robust impact evaluations into conservation. We hope this article will improve the quality of evaluations and help direct future research to continue to improve the approaches on offer.

## Abstract 2

*Article Impact statement*: The article identifies 3 steps in using matching in conservation impact evaluation, highlighting best practice and areas for improvement.

## Abstract (es)

Emparejamiento Estadístico para la Ciencia de la Conservación

**Resumen**

Hay más conciencia sobre la necesidad de evaluaciones de impacto más rigurosas y las técnicas de emparejamiento estadístico se están usan con más frecuencia para evaluar los impactos de intervenciones de conservación. Las técnicas de emparejamiento pueden ser herramientas poderosas si son utilizadas correctamente, pero también pueden llevar a conclusiones erróneas. En este artículo describimos consideraciones importantes para realizar estudios de conservación que utilizan técnicas de emparejamiento estadístico. Identificamos tres pasos dentro de un análisis de emparejamiento. Primero, es importante desarrollar una teoría del cambio que considere las complejidades del mundo real y las posibles consecuencias indirectas para poder seleccionar unidades de tratamiento y controles. Segundo, es clave seleccionar las covarianzas y la estrategia de emparejamiento apropiadas. Tercero, evaluar la calidad del emparejamiento a través de una serie de revisiones. El segundo y el tercer paso pueden ser repetidos y deben concluirse antes de que se exploren los resultados. Evaluaciones de impacto de intervenciones de conservación podrían mejorarse con una mejor planeación de las evaluaciones junto con la intervención, una mejor integración de los métodos cualitativos, mejor consideración de consecuencias indirectas a grandes escalas espaciales y un incremento en la publicación de planes pre‐analíticos. La implementación de estas mejoras requerirá de un compromiso más serio por parte de científicos de la conservación, y de practicantes y financiadores. Esperamos que este artículo mejore la calidad de evaluaciones y ayude a guiar futuras investigaciones e intervenciones de conservación.

## 摘要

目前, 已经有越来越多的人认识到需要对保护的效果进行稳健的评估, 在评估保护措施的影响时也越来越多地应用了统计匹配方法。匹配方法若使用得当可成为有力工具, 但也可能带来潜在陷阱。本研究概述了在保护科学中使用匹配方法时的重要注意事项和最佳实践。我们确定了匹配分析的三个步骤。首先是提出清晰的变化理论来指导实验组和控制组的选择, 其中要考虑到现实世界的复杂性和潜在的溢出效应。接下来, 应选择合适的协变量和匹配方法。最后, 需通过一系列的检查来评估匹配的质量。第二步和第三步可以重复进行, 但要在输出匹配结果前完全确定。在实施保护措施的同时加强评估规划、更好地整合总结定性方法、考虑更大空间尺度的溢出效应, 以及更多地公开发表预分析计划, 将有助于改善未来的保护效果评估体系。实施以上改进措施还需要保护科学家、实践者和资助者的认真参与, 推动保护效果的稳健评估纳入保护工作的主流。我们希望本文有助于提升保护效果评估的质量, 并帮助指导今后的研究以进一步改进现有的方法。**【翻译: 胡怡思; 审校: 聂永刚】**

## Introduction

There have been numerous calls for conservation science to provide a stronger evidence base for policy and practice (Pullin & Knight 2001; Sutherland et al. 2004; Baylis et al. 2016). Rigorous impact assessments of conservation interventions is vital to prevent wasting conservation resources (Ferraro & Pattanayak 2006) and tackling rapid biodiversity loss. Although the importance of establishing counterfactuals (what would have happened in the absence of an intervention) to generate more precise and less biased estimates of conservation impacts is increasingly recognized (Baylis et al. 2016), robust impact evaluations remain limited in number and scope (Schleicher 2018).

It is seldom feasible, or even desirable, to randomly implement conservation interventions for ethical, logistical, and political reasons. Experimental evaluations are therefore likely to remain rare (Baylis et al. 2016; Pynegar et al. 2018; Wiik et al. 2019). However, methodological advances to improve causal inference from nonexperimental data have helped to better attribute conservation impacts (Ferraro & Hanauer 2014a). These methods emulate experiments by identifying treatment and control groups with similar observed and unobserved characteristics (Rosenbaum & Rubin 1983; Stuart 2010). Among the range of nonexperimental approaches available for impact evaluations, each with their strengths and weaknesses (Table 1), matching approaches are playing an increasingly important role in conservation science (e.g., Andam et al. 2008; Nelson & Chomitz 2011; Naidoo et al. 2019).

**Table 1:** Pros and cons of commonly used nonexperimental, quantitative impact evaluation approaches in conservation

Method	When used	Pros	Cons
Matching*	baseline information on confounding factors (those affecting both selection of treatment and outcomes) available for both treatment and control units (e.g., Andam et al. 2008)	relatively few data requirements; lends itself to integration with other approaches when used as a data preprocessing step	assumes balance in observable covariates reflects balance in unobserved covariates (i.e., there are no unobserved confounders)
Before‐after‐control‐impact (difference‐in‐difference)	data before and after treatment implementation can be collected from replicated treatment and control units (e.g., Pynegar et al. 2018)	controls for time invariant variables and variables that change over time but affect both treatment and control groups equally	assumes a parallel trend in outcome between treatment and controls (confounding factors are those affecting treatment assignment and changes in outcome over time)
Regression discontinuity	selection of the intervention follows a sharp assignment rule (e.g., participants above a certain threshold are selected for treatment [Alix‐Garcia et al. 2018])	strong causal inference	outcomes calculated only for units close to the cutoff (i.e., data from only a small subgroup of units are used)
Instrumental variables	treatment assignment correlated with error term (endogeneity); a third variable (the instrument) correlated with treatment but uncorrelated with the error term can be used instead of the treatment (e.g., Liscow 2013)	helps overcome endogeneity	suitable instruments can be hard to find
Synthetic control	intervention has only occurred in a single unit of observation; information from a potential pool of controls can be synthesized to generate a single artificial counterfactual (e.g., Sills et al. 2015)	can be conducted when large numbers of treatment units are not available	credibility relies on a good prior to implementation fit for outcome of interest between treated unit and synthetic control

^*^Matching can be used to identify control units for comparison with treatment units as a method for impact evaluation, but is often used to improve the rigor of other approaches. For example, matching can be used to select control units for difference‐in‐differences analyses.

Matching comprises a suite of statistical techniques aiming to improve causal inference of subsequent analyses. They do so by identifying control units that are closely matched to treatment units according to predefined measurable characteristics (covariates) and a measure of similarity (Gelman & Hill 2007; Stuart 2010). Selecting comparable units of analysis (e.g., sites, individuals, households or communities) is important when conservation interventions are not assigned randomly. This is because units exposed to the intervention (treatment units), and those not exposed (control units) can differ in characteristics that influence the allocation of the treatment (i.e., where an intervention occurs, or who receives it) and the outcome of interest (e.g., species population trends, deforestation rates, changes in poverty levels). These characteristics are commonly referred to as confounding factors. For example, habitat conditions before an intervention can influence both the likelihood of the intervention being carried out in a specific location and habitat condition after the intervention's implementation.

Matching has 2 main applications in impact evaluation. First, where researchers seek to evaluate the impact of an intervention post hoc, matching can reduce differences between treatment and control units, and help isolate intervention effects. For example, when examining protected area (PA) effects on deforestation, distance from population centers (remoteness) is a likely confounder: remote sites tend to be more likely designated as protected and less prone to deforestation because they are harder to reach (Joppa & Pfaff 2009). Second, matching can be used to inform study design and data collection prior to the implementation of an intervention. For example, to evaluate how a planned conservation intervention affects local communities, matching can be used to identify appropriate control and treatment communities to monitor effects before and after the intervention's implementation (Clements et al. 2014).

Matching is a powerful statistical tool, but not a magic wand. The strengths and weaknesses of matching relative to alternative methods should be considered carefully, and its use optimized to maximize the benefits. Given the rapid rise in the use of matching approaches in conservation science, there is an urgent need for reviewing best practices and bringing together the diverse technical literature, mostly from economics and statistical journals (Imbens & Wooldridge 2009; Abadie & Cattaneo 2018), for a conservation science audience. The few existing related articles targeted at a conservation audience have focused on the conceptual underpinnings of impact evaluations (Ferraro & Hanauer 2014a; Baylis et al. 2016) without providing specific methodological insights. We addressed this gap by providing an overview of matching and key methodological considerations for the conservation science community. We did so by drawing on the wider literature and our own collective experience with matching in conservation impact evaluations. We focused on important considerations when using matching, outlined best practices, and highlighted key methodological issues that deserve further attention and development.

## Important Considerations When Using Matching in Conservation Impact Evaluation

### Three Key Steps

**Figure 1:**
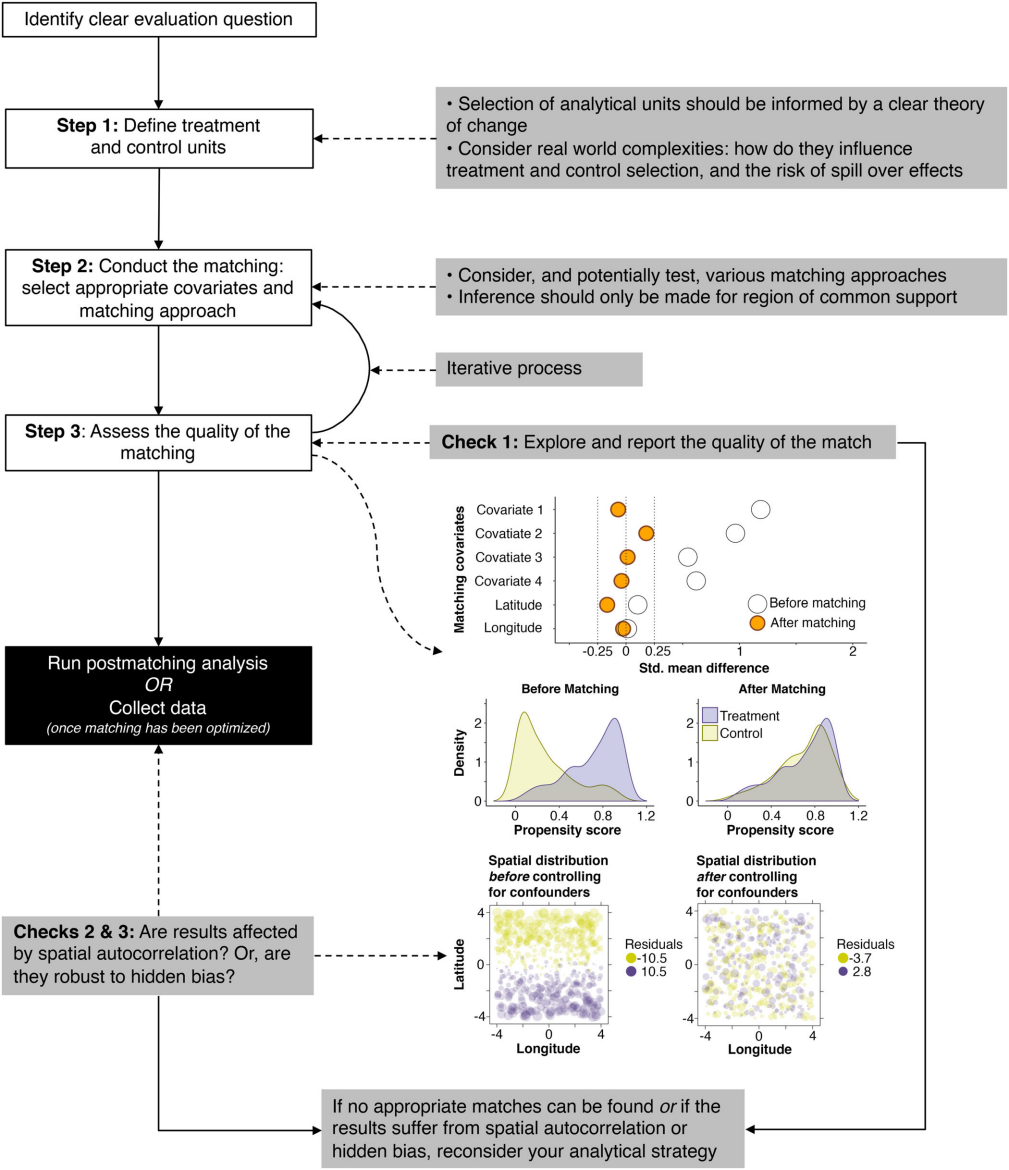
Visual representation of the suggested workflow, including key steps of a matching analysis, potential checks (see Table 2), and visual diagnostics of the matching process.

As with any statistical analysis, matching studies require careful design (Stuart 2010; Ferraro & Hanauer 2014a). We identify 3 main steps for a matching analysis (Fig. 1). The first step involves identifying units exposed to the treatment and those not. The second step consists of selecting appropriate covariates and the specific matching approach. The third step involves running the matching analysis and assessing the quality of the match (Table 2). Steps 2 and 3 should be repeated iteratively until the matching has been optimized. Only then should the matched data be used for further analysis. Doing so is important in post hoc analyses to avoid selecting a matching approach that produces a desired result (Rubin 2007). We elaborate on key considerations involved in each step (Fig. 1) below.

**Table 2:** Example diagnostics for the checks (suggested in Fig. 1) in a matching analysis to assess the quality of the matching and robustness of the postmatching analysis

	Example diagnostic	Explanation and purpose	Example visualizations
Check 1: balance	mean values and standardized mean differences before and after matching	test whether differences among treatment and control populations are meaningful. Compare covariate means and deviations for treatment and control units (before and after matching) to assess whether matching has improved balance (similarity between treatment and control units). After matching, mean covariate values should be similar and the standardized mean difference should ideally be close to 0. Standardized mean values of <0.25 are often deemed acceptable, but thresholds of 0.1 are more effective at reducing bias (Stuart 2010; Stuart et al. 2013).	love plots and propensity score distributions before and after matching (Fig. 1) (Oldekop et al. 2019)
Check 1: spatial autocorrelation	Moran's *I* and spatial distribution of postmatching analysis residuals	Moran's *I* values of the postmatching analysis should not be significantly different from 0 to demonstrate low levels of spatial autocorrelation. Plotting the spatial distribution of postmatching analysis residuals can help visualize whether there is a spatial pattern to the error term.	correlograms, semivariograms and bubble plots (Fig. 1) (Oldekop et al. 2019)
Check 3: hidden bias	Rosenbaum bounds	assess sensitivity of postmatching estimate to presence of an unobserved confounder. Rosenbaum bounds help determine how much an unobserved covariate would have to affect selection for treatment to invalidate the postmatching result (Rosenbaum 2007).	amplification plots (Rosenbaum & Silber 2009)

### Defining Treatment and Control Units (Step 1)

#### A NEED FOR A THEORY OF CHANGE TO MAKE EVALUATION POSSIBLE

The strength of the causal inference in observational studies relies on a clear understanding of the mechanism through which interventions influence outcomes of interest. Rival explanations should be carefully considered and, if possible, eliminated. Therefore, although impact evaluation is an empirical exercise, it requires a strong theory‐based explanation and model of the causal pathways linking the intervention to the outcomes of interest (Ferraro & Hanauer 2014b). This theoretical model is often referred to as a theory of change, causal chain, or logic model. It comprises a theoretical understanding of how a treatment interacts with the social‐ecological system it is embedded in (Qiu et al. 2018). This understanding is required to successfully argue that a causal pathway runs from the intervention to the outcome of interest (and not vice versa). For example, the expansion of a PA network might lead to the development of tourism infrastructure, which might also result in poverty reduction (Ferraro & Hanauer 2014b; den Braber et al. 2018). However, causality could run in the opposite direction: the development of tourism infrastructure close to a PA might be the outcome of reduced poverty as local communities invest revenue.

#### ACCOUNTING FOR REAL‐WORLD COMPLEXITY

**Figure 2:**
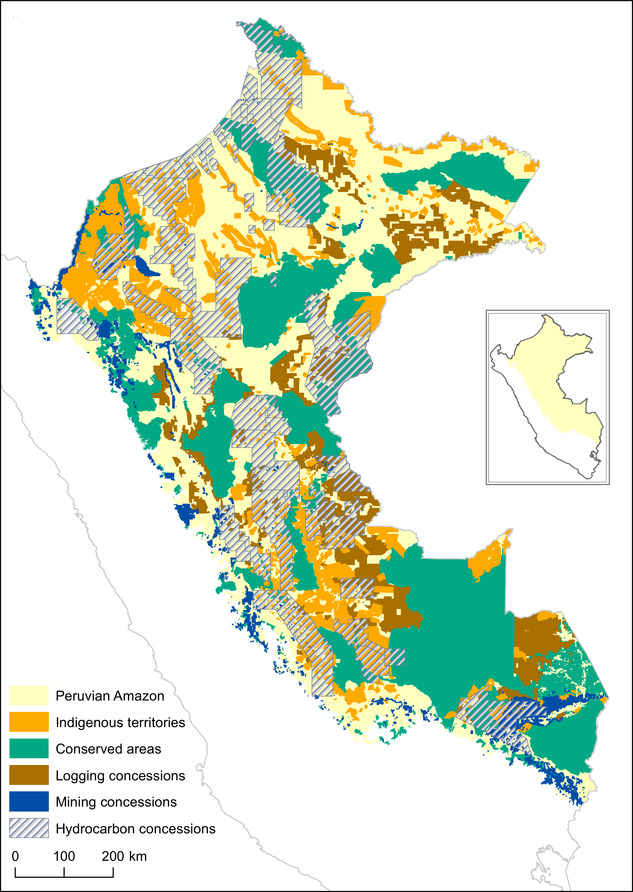
Main land‐use designations in the Peruvian Amazon in 2011 to 2013 (inset: Peru). Conserved areas include government‐controlled protected areas, conservation concessions, ecotourism concessions, concessions of nontimber forest products, and territorial reserves.

Conservation interventions are seldom implemented in simple settings where the impacts of 1 intervention can be easily separated from others. A thorough understanding of the study area and context is essential for identifying appropriate treatment and control units. Typically, conservation interventions are implemented in a landscape where potential treatment and control units have been exposed to a range of different interventions. Spatially explicit data sets, identifying where interventions have been implemented, are not uniformly available across space: spatial information for some interventions is much more readily available than for others (Oldekop et al. 2019). Teasing apart the effects of specific interventions can therefore be challenging. In the Peruvian Amazon, for example, there are few land areas with no formal or informal land‐use restrictions and the land‐use designations often overlap (Fig. 2). This hinders isolation of a particular treatment type (e.g., government‐controlled PAs or conservation concessions [Fig. 2]) and identification of appropriate control units (e.g., unprotected land without land‐use restrictions [Fig. 2]). Indeed, the few matching studies that have accounted for differences between land‐use restrictions show that the degree to which conservation interventions can be considered effective is influenced by how control areas are defined and selected (Gaveau et al. 2012; Schleicher et al. 2017). Conservation impact assessments could be improved by being more explicit about what the alternative land uses to the conservation interventions are and why specific controls were selected.

#### CONSIDERING SPILLOVER IN THE SELECTION OF CONTROLS

A central assumption in matching studies is that the outcome in 1 unit is not affected by the treatment in other units (Rubin 1980). However, this assumption does not always hold. There are many situations where outcomes in treatment units may spillover and affect outcomes in control units, either positively or negatively (Ewers & Rodrigues 2008; Baylis et al. 2016). For example, increased fish population in no‐take zones might spillover into adjacent unprotected habitats, a case of positive spillover that is part of the design of no‐take marine PAs. This would mask the positive impact of the intervention by reducing the difference between treatment and potential control units. In addition, fishing effort may be displaced from a no‐take zone into potential control areas (negative spillover). One might thus wrongly conclude that the intervention was successful, despite there being no overall reduction in fishing effort. In studies evaluating the impact of PAs on deforestation, negative spillovers (also called *leakage*) have usually been accounted for by excluding buffer zones around treatment areas, so that they cannot be included as controls (Andam et al. 2008). However, leakage effects can vary across landscapes (Robalino et al. 2017) and take place over larger geographical scales, which have so far not been accounted for in matching studies.

### Selecting Covariates and Matching Approach (Step 2)

#### SELECTING MATCHING COVARIATES INFORMED BY THE THEORY OF CHANGE

A key assumption in nonexperimental studies is that selection for treatment should be independent of potential outcomes (known as the conditional ignorability assumption [Rosenbaum & Rubin 1983]). If factors affecting treatment assignment can be ignored, all confounding factors should have been controlled for, and the study should not suffer from hidden bias (i.e., not be very sensitive to potential missing variables). Therefore, matching analyses should ideally include all covariates likely to impact both the selection to the treatment and the outcome of interest (e.g., remoteness, as how remote a piece of land will affect the likelihood of it being designated as PA and also deforested). Researchers should thus carefully consider which covariates are likely related to the outcome. It is better to err on the side of caution by including a covariate if the researcher is unsure of its likely role as a confounder. However, it is important that no variables likely to have been influenced by the outcome of interest are used as part of the matching process (Stuart 2010), so matching should only include variables predating the intervention or time‐invariant variables. Creating a table of all possible confounding factors that shows how they relate to the selection and outcome variables can help organize this process (e.g., Schleicher et al. 2017). Running regression analyses prior to matching or plotting the results of a principal component analysis (PCA) can also inform covariate selection. A PCA can help visualize how treatment and outcome relate to the selected covariates by showing which combination of covariates explains the outcomes observed in different units of analysis and whether treatment and outcome have similar patterns (Eklund et al. 2016).

#### CAREFUL SELECTION AND IMPLEMENTATION OF THE MATCHING APPROACH

There are various matching approaches, all with strengths and weaknesses. It is difficult to assess a priori which method is the most appropriate for a given study. Thus, testing a suite of different matching methods to evaluate which produces the best balance (step 3 in Fig. 1), instead of relying on any 1 method, can be useful (e.g., Oldekop et al. 2018). Matching approaches include Mahalanobis, propensity score, genetic, and full matching (Stuart 2010; Iacus et al. 2012; Diamond & Sekhon 2013). Mahalanobis and propensity score matching are particularly commonly used in conservation science, and there is growing interest in the use of genetic matching. Mahalanobis matching calculates how many standard deviations a unit is from the mean of other units (e.g., Rasolofoson et al. 2015). In contrast, propensity score matching combines all covariates into a single distance measure that estimates the probability of units receiving the treatment (e.g., Carranza et al. 2013). Genetic matching automates the iteration process (Diamond & Sekhon 2013) by optimizing balance diagnostics, rather than mean standardized distance (e.g., Hanauer & Canavire‐Bacarreza 2015). Full matching uses a propensity score to match multiple control units to treatment unit and vice versa. It is particularly well suited when analyzing data sets with similar number of treatment and control units (e.g., Oldekop et al. 2019). The development and testing of matching approaches remains an active research area with some strongly arguing for 1 method over another (King & Nielsen 2019).

Each of these methods can be configured in multiple ways, requiring a series of additional decisions, including about treatment to control ratio, replacement of control units, weighting, setting calipers, the order of selecting matches, and exact matching. First, for the ratio of treatment to control units used during matching, one must decide whether to apply 1‐to‐1 matching or to match 1 treatment unit to several control units. Second, regarding the replacement of control units, the choice is whether control units can be used multiple times or not (i.e., match with or without replacement). Third, the relative importance of retaining as many treatment units or control units in the analysis as possible, and hence the relative weighting of different units, must be considered carefully. Some approaches apply sampling weights to give more importance to certain units and to adjust for unbalanced data sets. Fourth, one must decide whether to set bounds (called *calipers*) on the degree of difference between treatment and control units. Fifth, one can set the order in which matches are selected (e.g., at random or in a particular order) (Lunt 2014). Finally, one must decide whether to retain only units with the exact same covariate value (called *exact matching*) or not. Exact matching using continuous covariates typically results in many treatment units being excluded because no control units with identical values are found. This can increase bias because data is being systematically discarded. It is thus better suited for categorical variables.

#### BASING INFERENCE ONLY ON THE REGION OF COMMON SUPPORT

In some cases, treatments may be so closely interlinked with potential confounders that no good matches exist. For example, if intact habitat remains only on mountain tops and all mountain tops are protected, it would be impossible to separate the contribution of location from that of the intervention itself because there are no controls with similar habitat available that are not protected (Green et al. 2013). Matching therefore depends on a substantial overlap in relevant covariates between units exposed to the intervention and potential controls. This overlap is known as the region of common support. An assessment of common support early on in the matching process can be a good filter to determine whether matching will be useful. When using the propensity score, it is simple to discard potential control units with scores outside the range of the treatment group. Visual diagnostics, including the propensity score distribution, are a simple and robust way of diagnosing any challenges with common support (Lechner 2000; Caliendo & Kopeinig 2008) (Fig. 1 & Table 2). Where many potential control units need to be discarded, it can be helpful to define the discard rule based on 1 or 2 covariates rather than the propensity score (Stuart 2010). If many treatment units must be discarded because no appropriate control units can be found, the research question being answered by the analysis is likely to be different from the one asked to begin with. This needs to be acknowledged. In some cases, it will simply not be possible to use matching to evaluate the impact of an intervention on an outcome of interest, requiring the use of alternative quantitative or qualitative methods (e.g., Green et al. 2013).

### Assessing the Quality of the Matching (Step 3)

#### EXPLORING AND REPORTING QUALITY OF THE MATCH ACHIEVED

Matching provides no guarantee that biases have been sufficiently addressed. It is therefore important to assess the quality of the match and to report relevant statistics (Fig. 1 & Table 2). In fact, an advantage of using matching rather than standard regression is that it highlights areas of the covariate distribution where there is not sufficient common support between treatment and control groups to allow effective inference without substantial extrapolation (Gelman & Hill 2007). When assessing the performance and appropriateness of a match, 3 key features should be assessed and reported: first, how similar are the treatments and controls after matching (covariate balance); second, how similar is the prematch treatment to the postmatch treatment (large dissimilarities can potentially increase bias); and third, the number of treatment units that were matched and discarded during matching. In addition, when matching is done with replacement, it is prudent to check the selection rate of matched controls, to ensure that there is no oversampling of specific controls. The best matching method will be the one that keeps the postmatch treatment as similar to the prematch treatment as possible, while ensuring maximum similarity between postmatch treatment and control units, and removing the least number of observations in the process. The proportion of covariates that have met a user‐specified threshold for balance and the covariate with the highest degree of imbalance are effective indicators in diagnosing imbalance and potential bias (Stuart et al. 2013). Standard tests and visualizations that explore match quality have been widely published in the statistical, economics, health, and political science literatures (e.g., Rubin 2001; Harris & Horst 2016). It is useful to combine both numeric and visual diagnostics (examples in Table 2) (Caliendo & Kopeinig 2008; Stuart 2010; Harris & Horst 2016).

A central assumption underlying the use of matching approaches is that any difference between treatment and control populations remaining after matching are due to treatment effects alone. Validating this assumption rests on a robust theory of change and a careful selection of covariates. However, even if all known sources of potential bias have been controlled for, unknown mechanisms might still confound either treatment or outcomes. Checks to assess whether postmatching results are sensitive to potential unmeasured confounders (e.g., Rosenbaum bounds [Rosenbaum 2007]) allow one to evaluate the amount of variation that an unmeasured confounder would have to explain to invalidate the results.

#### CONSIDERING THE ROBUSTNESS OF MATCHING RESULTS TO SPATIAL AUTOCORRELATION

Conservation interventions, and most data used to assess their impacts, have a spatial component. A key assumption of many statistical tests is that units of observation are independent from each other (e.g., Haining 2003; Dormann et al. 2007). Yet, this assumption is easily violated when using spatial data: units of observation that are closer together in space are often more similar to each other than units of observation that are further apart. Such spatial dependency, referred to as spatial autocorrelation (SAC), is often not discussed or explicitly tested for in conservation matching studies, despite being a well‐recognized phenomenon (Legendre 1993; Dormann et al. 2007). While it is unclear how matching affects SAC, SAC can clearly affect impact estimations. For example, studies modeling deforestation have shown that the spatial coordinates of a data point are among the top predictors of deforestation (Green et al. 2013; Schleicher et al. 2017). Some matching studies in the conservation literature have acknowledged the potential resulting bias and attempted to account or test for any potential effects linked to the spatial sampling framework (e.g., Carranza et al. 2013; Schleicher et al. 2017; Oldekop et al. 2019). We call for increased attention to SAC when evaluating place‐based interventions. Steps to test for SAC include Moran's *I* tests, semivariograms, correlograms, and spatial plots of model residuals (Schleicher et al. 2017; Oldekop et al. 2019). These could be used to test for SAC of postmatching analyses and treatment assignment (e.g., by testing SAC of propensity score models). The SAC could also be tested separately in the treatment and control groups before and after matching. If significant SAC remains after matching, it would be a strong indication that it needs to be accounted for in any postmatching regression, something that could be confirmed through inspection of spatial patterns of model residuals (Dormann et al. 2007; Zuur et al. 2009; Oldekop et al. 2019).

### Postmatching Analyses

Matching is often used as a data preprocessing step (Ho et al. 2007). If matching perfectly reduces the difference between treatment and control units to 0, or the residual variation is close to random and uncorrelated with treatment allocation and the outcome of interest, then the average treatment effect can be measured as the difference in the outcome between treatment and control units. However, in most instances matching reduces—but does not eliminate—differences between treatment and control units. It is often followed by regression analyses to control for any remaining differences between treatment and control units (Imbens & Wooldridge 2009). Where longitudinal panel data are available, matching can be combined with a difference‐in‐difference research design (e.g., Jones & Lewis 2015) (Table 1). Combining matching with other statistical methods in this way tends to generate treatment‐effect estimates that are more accurate and robust than when using any 1 statistical approach alone (Blackman 2013).

## Moving Forward

The increasing use of matching approaches in conservation science has great potential to rigorously inform what works in conservation. However, while matching approaches are a powerful tool that can improve causal inference, they are not a silver bullet. We caution against using matching approaches without a clear understanding of their strengths and weaknesses. Looking to the future, we highlight clear avenues for improving the use of matching in conservation studies. This includes developing robust theories of change, incorporating real‐world complexities, careful selection of matching variables and approaches, assessing the quality of matches achieved, and accounting for SAC. Conservation impact evaluation would benefit from increased evaluation planning alongside conservation interventions, better integration of qualitative approaches with quantitative matching‐based methods, further consideration of how spillover effects should be accounted for, and increased publication of preanalysis plans. We explored each of these in turn.

Post hoc evaluations are often necessary in conservation because there is a pressing policy need to explore the impacts of past interventions. However, there are limits to what statistical analyses can do post hoc to overcome problems in the underlying study design of an impact evaluation (Ferraro & Hanauer 2014a). More integration of impact evaluations within intervention implementations is needed to address and account for biases in where interventions are located. Occasionally, this may provide the opportunity for experimental evaluation (Pynegar et al. 2018; Wiik et al. 2019). More commonly, where this is not possible or desirable, good practice should be to explore and consider potential controls using matching from as early as possible. Innovative funding is needed to allow researchers to work alongside conservation practitioners throughout their intervention to incorporate rigorous impact evaluation from the start (Craigie et al. 2015).

Matching does not provide certainty about causal links and on its own is unlikely to provide insights into the mechanism by which an intervention had an impact. This highlights the importance of making use of the diverse set of evaluation approaches and data sources available. This includes the important, but often overlooked, contribution that qualitative data can make to impact evaluation and counterfactual thinking. For example, incorporating qualitative data can provide depth in understanding, identify hypotheses, and help find potential reasons underlying the effect of an intervention. Process tracing, realist evaluation, assessment of exceptional responders, and contribution analyses are all suited for exploring the mechanisms by which an intervention led to an outcome (Collier 2011; Lemire et al. 2012; Westhorp 2014; Meyfroidt 2016; Post & Geldmann 2018). Qualitative comparative analysis can also be useful for exploring what factors needed to be present to achieve successful outcomes or how impacts vary among different groups and circumstances (Korhonen‐Kurki et al. 2014).

There are remarkably few explicit assessments of the importance of spillover effects beyond intervention boundaries at different spatial scales (Pfaff & Robalino 2017). While impact evaluations on deforestation rates commonly avoid selecting control pixels from a predefined buffer area around an intervention, the size of the buffer is seldom based on a clear justification. We know of no matching studies that explicitly account for spillover effects over larger spatial scales. This is despite the need to account for spillovers to assess whether a net reduction in conservation pressure has taken place, instead of simply displacing it elsewhere (Pfaff & Robalino 2012). For example, stronger implementation of logging rules in 1 region of Brazil shifted pressures to other regions (Dou et al. 2018) and China's national logging bans mean that timber demand is being met through imports from Indonesia (Lambin & Meyfroidt 2011). Many factors complicate the ability to account for these effects over large spatial scales, including demand and supply dynamics, feedback cycles, and behavioral adaptation (Ferraro et al. 2019). Accounting for such factors will require further collective, interdisciplinary thinking and methodological developments.

There is a push for researchers in a number of fields to publish preanalyses plans (e.g., Nosek et al. 2018), which lay out hypotheses identified a priori and proposed analyses before the effects are assessed (Bauhoff & Busch 2018). The aim of preanalyses plans is to reduce the risk of HARKing (hypothesising after results are known [Kerr 1998]). As there are many potential acceptable ways to select appropriate matches, there are benefits in publishing the matching and planned analysis before carrying it out.

Given continuous loss of biodiversity despite considerable conservation efforts, there is an urgent need to take impact evaluations more seriously, learn from other disciplines, and improve our practices as a conservation science community. The increasing interest in the use of counterfactual approaches for evaluating conservation impacts is therefore a very positive development. There is an important role for conservation practitioners, funders, and academics to encourage this development and to mainstream rigorous impact evaluations into conservation practice. Furthermore, there is certainly a need to increase the capacity of conservation scientists and practitioners in both the conceptual and technical challenges of impact evaluation, including by incorporating impact evaluation and counterfactual thinking in postgraduate training of future conservationists. We hope our article will help improve the general quality of evaluations being undertaken and direct future research to continue to improve the approaches currently on offer.
